# Supplementary material for: Compressed ultrahigh-speed single-pixel imaging by swept aggregate patterns
Source: Nat Commun. 2022 Dec 22;13:7879. doi: 10.1038/s41467-022-35585-8 (PMC9780349; doi:10.1038/s41467-022-35585-8)
Supplement: Supplementary file 1 — Supplementary Information [file 41467_2022_35585_MOESM1_ESM.pdf]

Supplementary Material

Compressed ultrahigh-speed single-pixel imaging by swept aggregate patterns

Patrick Kilcullen, Tsuneyuki Ozaki, and Jinyang Liang*

Centre Énergie Matériaux Télécommunications, Institut National de la Recherche Scientifique, Université du Québec, 1650 boulevard Lionel-Boulet, Varennes, QC J3X 1P7, CANADA.

*Corresponding author: jinyang.liang@inrs.ca

Supplementary Note 1: Details of system setup

A detailed illustration of the experimental setup of single-pixel imaging accelerated via swept aggregate patterns (SPI-ASAP) is shown in Supplementary Fig. 1a. Light from a continuous-wave laser source (200 mW power, 671 nm wavelength, MRL-III-671, CNI Laser) illuminates a high-speed projection module, whose core components are a polygonal mirror (Gecko-45-HSS, Precision Laser Scanning) and a digital micromirror device (DMD, AJD-4500, Ajile Light Industries). This high-speed polygonal mirror consists of 16 planar facets and has scanning rates at 2.7–12 kHz (corresponding to 10,000–45,000 RPM). It is used in a double-reflection arrangement [1] that increases optical efficiency by allowing illumination to be concentrated on only the parts of the DMD surface that are transmitted by the system. In particular, the laser beam is focused by the lens L1 (30 mm focal length) to a moving facet of the polygonal mirror placed at the lens' focal plane. The reflected beam is collimated by lenses L2 (75 mm focal length) and L3 (125 mm focal length) that are separated by 63.2 mm (Supplementary Fig. 1b) so as to produce an effective focal length appropriate for de-scanning by lens L4 (75 mm focal length). In this way, the mirror rotation is transferred to the lateral translation of the beam with its chief ray parallel to the optical axis (detailed in Methods). Then, the beam is reflected by two flat mirrors M1 and M2. A small portion of the beam passes beyond the edge of M2 and enters Iris 1 so that the "start-of-scan" photodiode (SOS-PD, DET100A2, Thorlabs) triggers the display of a new aggregate pattern that is pre-stored by the DMD. The rest of the beam scans across the DMD surface with an incident angle of $\theta = \sim 24°$.

The diffracted beam from the DMD, which is spatially modulated by the displayed aggregate pattern, is imaged to a slit via a 4-f imaging system consisting of lenses L4 and L5 (75 mm focal lengths) as well as a moving facet of the polygonal mirror placed at L4's focal plane. Iris 2, positioned close to the polygonal mirror, selects the strongest diffraction order. The width

of the slit determines the encoding patterns by optically selecting the sub-regions in the aggregate pattern. The intermediate image at the slit is further relayed by lens L6 (100 mm focal length) to the object with a varied magnification of 1.5–14×. Along the beam path, an adjustable neutral density filter (NDF) controls the illumination intensity while sending the reflected beam to the "time coding" photodiode (TC-PD, DET36A2, Thorlabs). Depending on the operational mode, the transmitted or reflected light of the object is focused by a condenser lens (CL) to the "signal acquisition" photodiode (SA-PD, DET36A2, Thorlabs) whose output waveforms are amplified by a transimpedance amplifier (OPA1S2384EVM, Texas Instruments).

Supplementary Note 2: System timing and data registration

The SOS-PD and TC-PD are used in the synchronization and data registration of SPI-ASAP. Signal path and timing diagrams are illustrated in Supplementary Fig. 3. As shown in Supplementary Fig. 1, each mirror scan produces a pulsed signal from the SOS-PD. From these signals, a delay generator (DG645, Stanford Research Systems) generates a per-scan timing datum via triggering on the rising edge of a pre-set voltage threshold. Outputs from the delay generator then trigger the refresh of DMD patterns as well as the acquisition of data using a digitizer card (ATS9350, AlazarTech) that interfaces with a computer. Data from both the TC-PD and SA-PD are synchronously stored as waveforms consisting of 1024 points of 12-bit measurements sampled at a rate of 20 MHz.

SA-PD waveforms are registered with the corresponding sub-areas in each aggregate pattern by using three special-purpose registration patterns appended to the beginning of the pre-stored DMD pattern sequence. Illustrated examples of SOS-PD, TC-PD, and SA-PD waveforms corresponding to these registration patterns are shown in Supplementary Fig. 3b. The first pattern (marked as (i) in Supplementary Fig. 3b) is all dark and serves as a datum for matching the waveforms measured by the TC-PD and SA-PD to the aggregate pattern sequence. The second and third patterns (marked as (ii) and (iii) in Supplementary Fig. 3b) consist of "On" DMD pixels in the initial and final encoding pattern sub-regions of the aggregate pattern, respectively. In SPI-ASAP, these patterns produce triangular TC-PD waveforms, whose peak locations supply timing offsets t_1 and t_2 that identify the positions of the initial and final deployed masking patterns (see

an example labeled by (iv) in Supplementary Fig. 3b) in the SA-PD waveform. Sampling of bucket signal values then follows by linear interpolation of sample locations within the $[t_1, t_2]$ interval.

Supplementary Note 3: Performance comparison for SPI using polygonal mirror scanning

Compared to the existing work on scanning-enhanced SPI [2,3], the design used in SPI-ASAP has several advantages. In the SPI system demonstrated by Wang et al. [2], a pair of galvanometer mirrors (GMs) were used to implement bi-directional sweeping of DMD-modulated patterns for compressed imaging at a speed of 42 fps and a frame size of 80×80 pixels. However, although the roles of the two GMs were functionally similar to the double-reflection arrangement of SPI-ASAP, the use of galvo-actuation limited the GM's scan rate to 200 Hz. In addition, the scanning geometry required the motion of the independent GMs to be calibrated and tightly synchronized. In another work, the SPI system demonstrated by Zhang et al. [3] achieved a higher scan rate by using a GM in tandem with a resonant scanning mirror that operated at 8 kHz. Sampling was thus achieved with the use of an optimized low-resolution DMD pattern that was scanned in a 2D fashion across the FOV. Although this modality achieved a modulation rate above 1 MHz, the fixed actuation frequency of the resonant scanning mirror ultimately limited the scan rate while still requiring both calibration and synchronization.

In contrast, the choice of a polygonal mirror for SPI-ASAP simultaneously addresses several limitations of designs based on GMs and resonant scanning mirrors. First, the ability to simultaneously deflect off of two separate mirror facets in the same device allows for scanning hardware to be consolidated, thus avoiding the synchronization of multiple mechanical devices. Second, because the angular speed of the facets on the polygon mirror remains constant in operation, motorization is simplified thus allowing for many-faceted polygons to achieve scan rates above 10 kHz. Scan rates may also be continuously varied with the rotational speed of the motor, thus increasing flexibility over resonant scanning mirrors whose period of oscillation is fixed. In addition, the time dependence of the scan angle for polygonal mirrors is linear, thus avoiding the need for precise calibration of control signals. Finally, by eliminating the deceleration period required by GMs and resonant scanning mirrors, polygonal mirrors allow for increased scan duty cycles, which may be optimized via the correct selection of facet count.

For the SPI-ASAP system, the use of off-the-shelf hardware dictated the use of a 16-sided mirror polygon. For aggregate patterns making use of the full DMD surface, this restriction resulted in a pattern deployment duty cycle of approximately 8%. Consequently, the rate of pattern modulation for SPI-ASAP is non-constant, with the current system compatible with roughly a 10× increase in polygon facets. To reflect the potential performance of SPI-ASAP with optimized facet count, modulation rates shown in Supplementary Table 1 are computed relative to the pattern deployment interval, thus reflecting the peak modulation rate during operation.

Supplementary Note 4: Comparison of conventional compressed-sensing methods with SPI-ASAP

To verify the performance of SPI-ASAP's reconstruction method, we compared image reconstruction between SPI-ASAP and two widely used compressive reconstruction algorithms implemented with the L1-Magic software library [4]. The first algorithm, termed "MinTV", is regularized based on the total variation (TV). The second algorithm, termed "MinDCT", is regularized from sparse representations using the basis of the discrete cosine transform (DCT) [5]. We define the "MinTV" and "MinDCT" reconstruction algorithms based on the following optimization programs found in the L1-Magic software package:

$$\underset{x}{\operatorname{argmin}}\ TV(\mathbf{x}) \quad \text{subject to} \quad \|S\mathbf{x}-\mathbf{y}\|_2 \le \epsilon\ , \qquad \text{(MinTV)}$$

$$\underset{x}{\operatorname{argmin}}\ \|\Psi\mathbf{x}\|_{\mathbf{1}} \quad \text{subject to} \quad \|S\mathbf{x}-\mathbf{y}\|_2 \le \epsilon\ , \qquad \text{(MinDCT)}$$

where $\mathbf{x}$ is the image to be recovered, $\mathbf{y}$ is the vector of bucket signals, S is the measurement matrix, ϵ is a user-specified parameter, and $\|\cdot\|_{\mathbf{1}}$ denotes the l_1-norm. For an image with a frame size of $p \times q$, the total variation $TV(\mathbf{x})$ is defined as

$$TV(\mathbf{x}) = \sum_{j=1}^{p-1}\sum_{j=1}^{q-1}\sqrt{[x(i+1,j)-x(i,j)]^2+[x(i,j+1)-x(i,j)]^2}\ , \qquad \text{(S1)}$$

where we write $x(i,j)$ to indicate the value of the pixel of $\mathbf{x}$ with coordinates (i,j). Finally, Ψ denotes the real orthogonal matrix that induces the DCT.

For quantitative comparison of the agreement between a reconstruction $\mathbf{x}$ and ground truth image $\hat{\mathbf{x}}$, we used both the peak signal-to-noise ratio (PSNR) and structural similarity index (SSIM) as metrics to compute reconstruction error [6,7]. The PSNR and SSIM are defined as

$$PSNR(\mathbf{x},\hat{\mathbf{x}}) = 10\log_{10}\left(\frac{(\max(\hat{\mathbf{x}}))^2}{(1/pq)\|\mathbf{x}-\hat{\mathbf{x}}\|_2^2}\right), \tag{S2}$$

and

$$SSIM(\mathbf{x},\hat{\mathbf{x}}) = \frac{(2\mu_1\mu_2 + C_1)(2\sigma_{12} + C_2)}{(\mu_1^2 + \mu_2^2 + C_1)(\sigma_1^2 + \sigma_2^2 + C_2)}. \tag{S3}$$

Here, $\max(\hat{\mathbf{x}})$ denotes the maximum intensity value of the ground truth image. μ_1 and μ_2 denote the mean intensity values of $\mathbf{x}$ and $\hat{\mathbf{x}}$, respectively. σ_1^2 and σ_2^2 denote the variances of the intensity values of $\mathbf{x}$ and $\hat{\mathbf{x}}$, respectively. σ_{12} denotes the covariance of $\mathbf{x}$ and $\hat{\mathbf{x}}$. The constants C_1 and C_2 are used to prevent indeterminate values of SSIM and were set to the conventional values $(0.01\max(\hat{\mathbf{x}}))^2$ and $(0.03\max(\hat{\mathbf{x}}))^2$, respectively [4].

Similar to other iterative reconstruction methods based on convex optimization, the selection of the user-defined parameter ϵ plays a crucial role in the performance of MinTV and MinDCT. For this numerical experiment, we selected ϵ based on a golden section search that first optimized the PSNR against the ground truth image. Thus, the results of the MinTV and MinDCT methods in Supplementary Fig. 4 could be interpreted as optimized with respect to the selection of ϵ.

We also measured the time overhead required for reconstructions provided by each method using a computer with an Intel® Core™ i5-8250U CPU (1.60 GHz) and 8 GB RAM. Input to each reconstruction method included identical measurement matrices and simulated bucket signals. All measurement matrices were composed in accordance with SPI-ASAP's aggregate pattern coding strategy. No noise was injected in this experiment.

The results of this comparison, shown in Supplementary Fig. 4, indicate that in terms of PSNR and SSIM, the performance of SPI-ASAP largely meets or exceeds that of the iterative methods, in particular outperforming them across both metrics for the case of sampling rates below 50%. This observation is qualitatively matched by the subset of image reconstruction results shown in Supplementary Fig. 4d. For the case of 100% sampling, we note highly accurate convergence across all methods as indicated by the pronounced maxima exhibited by the PSNR metric. Measurements of reconstruction time required for each algorithm (Supplementary Fig. 4c) provide the most striking comparison between SPI-ASAP and the conventional methods, with SPI-ASAP reconstructions exhibiting consistent running times 2–3 orders of magnitude below those of both conventional methods.

Supplementary Note 5: Details of high-speed imaging of incandescent filaments

The setup for acquiring optically filtered bucket signals is illustrated in Supplementary Fig. 5. The incandescent filament was placed at SPI-ASAP's image plane, which was in the front focal plane of lens L1 (75 mm focal length). Iris 1, positioned in the back focal plane of L1, blocked the majority of incandescent rays that were approximately collimated. Lens L2 (75 mm focal length), in a 4-*f* configuration with L1, relayed the image of the filament to the surface of a diffraction grating (600 lines per mm, GR13-0605, Thorlabs) from which the first diffraction order was selected. The grating was positioned on the focal plane of lens L3 (100 mm focal length), which produced a chromatically dispersed image of Iris 1 at Iris 2. Finally, 671 nm light chromatically selected by Iris 2 was focused by the condenser lens to the SA-PD.

The light bulbs used in the experiments were designed for low-power applications (rated 12 V, 18 W) and were commercially sourced. In each experiment, filament burn-out was initiated by the application of 24 V with a DC power supply. Imaging experiments were repeated several times for each case with consistent results, with representative videos being presented in Main Text and Supplementary Movies 4 and 5.

Supplementary Note 6: Details of ultra-high-speed imaging at 12 kfps

The timing and data registration scheme used for ultra-high-speed imaging with SPI-ASAP is illustrated in Supplementary Fig. 6. For this imaging arrangement, bucket signals are generated from a single displayed DMD pattern that remains static during the scanning of the polygonal mirror at its maximum speed of 12 kHz. The static DMD pattern is comprised of six jointly arranged aggregate patterns, each designed with a resolution of 11×13 pixels. Thus, this DMD pattern carried a total of 78 masking patterns corresponding to a sampling rate of 55%.

Data registration for the joint aggregate pattern proceeded similarly to the procedure described in Supplementary Note 2 with two exceptions. First, multiple intervals were used to derive bucket signals. Second, registration patterns were not displayed synchronously with the collection of data. The registration patterns, shown by insets (i) and (ii) in Supplementary Fig. 6, consisted of joint copies of the registration patterns appropriate for each segment of the joint

aggregate pattern. Waveforms for the TC-PD and SA-PD were sampled at 50 MHz with TC-PD waveforms being collected separately for each registration pattern. The locations of the six triangular peaks within each TC-PD waveform then supplied timing offsets $t_1^{(i)}$ and $t_2^{(i)}$ ($i = 1, \dots, 6$) that identified the positions of the initial and final encoding patterns deployed by each segment of the multi-aggregate pattern. Linear interpolation of sample locations within the six intervals $[t_1^{(i)}, t_2^{(i)}]$ ($i = 1, \dots, 6$) then determined bucket signal values from SA-PD waveforms collected during the experiment.

Video reconstruction was carried out in a one-to-one fashion with SA-PD waveforms, with the 78 bucket signals derived from each waveform leading to the reconstruction of individual frames in sequence. The time interval to acquire each frame was approximately 6.7 μs.

Supplementary Note 7: Investigation of spatial resolution of SPI-ASAP

To investigate the spatial resolution of SPI-ASAP, we imaged a resolution target (1951 USAF) at four rotation angles between 0° and 90°. The SPI-ASAP system had a frame size of 59×61 pixels across a FOV of size 5.5 mm (i.e., each pixel is 90 μm in size), which was the most typical setting used in our work. At each rotation position, the image was obtained as temporal averages of 89 consecutively collected frames. For frame reconstruction, spatial filtering was disabled via the choice of $T = 0$ in equations (S5) and (S6), which set the spatial filtering matrix V equal to the identity (see Supplementary Note 8). Line profiles of normalized intensity were then extracted that were tracked to individual elements of the resolution target over each rotation position.

Imaging results are illustrated in Supplementary Fig. 7. Evidence of a non-isotropic spatial resolution can be observed in the variation of line profile modulation depth with the rotation angle of the resolution target. Using the criterion of 5% contrast, we determined a horizontal resolution of 198.4 μm identified as the resolution target line width (Group 1, Element 3) corresponding to profile 2 shown in Supplementary Fig. 7a. Similarly, we determined a vertical resolution of 88.4 μm identified as the resolution target line width (Group 2, Element 4) corresponding to profile 2 shown in Supplementary Fig. 7b.

Two factors can be identified as contributing to the resolution anisotropy. First, to prioritize speed, SPI-ASAP's data registration procedure (see Supplementary Note 2) does not compensate for encoding pattern motion that occurs during intervals sampled for each bucket signal. For a scan

rate of 6.37 kHz and a modulation duty cycle of 8%, the time interval containing bucket measurements is of duration 0.08 / 6.37 kHz = 12.6 μs. Consequently, for 59×61-pixel imaging, a shift of one encoding pixel occurs after a period of 12.6 μs / 61 = 0.21 μs. Thus, with 20 MHz sampling of SA-PD waveforms, a pattern shift of ±0.12 encoding pixels occurs within the sampling interval. Second, the TC-PD waveforms exhibited a 16-period timing variation produced from minute errors in the facet geometry of the polygonal mirror. For high-speed imaging, this resulted in a timing variation of 0.19 μs, equivalent to ±0.45 encoding pixels. Although the extraction of appropriate timing offsets for data registration was stabilized by temporal averaging, timing variations affecting the registration of bucket signals is still capable of affecting reconstruction results via row-wise misalignment of data within the bucket signal matrix Y.

Supplementary Note 8: Details of matrix pre-computations for interpolation-based image reconstruction

Derivation of V

Let F denote the $q \times q$ matrix of the discrete Fourier transform (DFT) of order q, which is defined by $F_{i,j} = \omega^{-ij}$, where i and j range over $(0, \ldots, q-1)$, and ω is a primitive q^{th} root of unity. The inverse of this matrix can be computed by $F^{-1} = (1/q)\, F^*$, where F^* denotes the complex conjugate transpose of F. The row-wise spatial filtering of a matrix M can be written as

$$MV = \left(\tfrac{1}{q} F^* D F M^T\right)^T = M\left(\tfrac{1}{q} F D F^*\right) \ , \tag{S4}$$

where the matrix D is diagonal and selects the spatial frequencies retained from the DFT. For a symmetric low-pass filter whose cut-off frequency is denoted by T $(2T \leq q)$, the diagonal elements of D can be defined as:

$$D_{i,i} = \begin{cases} 0 & \text{if } \ T < i < q - T \\ 1 & \text{otherwise} \end{cases} \ , \tag{S5}$$

from which the elements of V can be computed as:

$$V_{i,j} = \tfrac{1}{q} F D F^* = \frac{1}{q}\left[1 + 2\textstyle\sum_{k=1}^{T} \cos(2\pi(i-j)k/q)\right] \ . \tag{S6}$$

Across our experiments, only mild low-pass filtering was used, with values of T selected to retain 70%–80% of the spectrum of the DCT.

Derivation of W

Column-wise interpolation was developed as a special case of piecewise cubic spline interpolation with cyclic boundary conditions. As illustrated in Supplementary Fig. 8, from the four data points shown, a cubic interpolating polynomial $f(x)$ can be specified for values $x_1 \leq x < x_2$, according to the boundary conditions

$$\begin{aligned} f(x_1) &= y_1 \ , \\ f(x_2) &= y_2 \ , \\ f'(x_1) &= g_1 \ , \text{and} \\ f'(x_2) &= g_2 \ , \end{aligned} \tag{S7}$$

where the values of g_1 and g_2 are chosen as the average slopes between the two line-segments that meet at positions x_1 and x_2, respectively, i.e.,

$$\begin{aligned} g_1 &= \frac{1}{2}\left(\frac{y_1 - y_0}{x_1 - x_0} + \frac{y_2 - y_1}{x_2 - x_1}\right) \ , \text{and} \\ g_2 &= \frac{1}{2}\left(\frac{y_2 - y_1}{x_2 - x_1} + \frac{y_3 - y_2}{x_3 - x_2}\right) \ . \end{aligned} \tag{S8}$$

For fixed values of $x_0, \ldots, x_3$ and x, values of the resulting interpolation function $y = f(x)$ are linear with respect to the y-coordinate data $y_0, \ldots, y_3$ and can be computed according to

$$y = H(x_0, x_1, x_2, x_3, x) \begin{bmatrix} y_0 \\ y_1 \\ y_2 \\ y_3 \end{bmatrix} \ , \tag{S9}$$

where $H(x_0, x_1, x_2, x_3, x)$ is defined as

$$H(x_0, x_1, x_2, x_3, x) = [x^3 \ \ x^2 \ \ x \ \ 1] \begin{bmatrix} x_1^3 & x_1^2 & x_1 & 1 \\ x_2^3 & x_2^2 & x_2 & 1 \\ 3x_1^2 & 2x_1 & 1 & 0 \\ 3x_2^2 & 2x_2 & 1 & 0 \end{bmatrix}^{-1} \begin{bmatrix} 0 & 1 & 0 & 0 \\ 0 & 0 & 1 & 0 \\ -\delta_1 & \delta_1 - \delta_2 & \delta_2 & 0 \\ 0 & -\delta_2 & \delta_2 - \delta_3 & \delta_3 \end{bmatrix} \ , \tag{S10}$$

and where $\delta_i = 1/[2(x_i - x_{i-1})]$.

The function $H(x_0, x_1, x_2, x_3, x)$ is now used to define a pre-computed matrix $\widehat{W}$ as follows. For imaging with resolution $p \times q$ and segmentation length L, the numbers u_k ($k = 0, \ldots L - 1$) are defined according to $u_k = d_{a+k}$ (see equation (8) in Main Text) where a is the index of the first aggregate pattern deployed in the data segment and the index $a + k$ is interpreted modulo p. We then define $\sigma(k)$ to be the permutation that sorts the u_k into strictly increasing

order: $u_{\sigma(0)} < \cdots < u_{\sigma(L-1)}$. By relabeling these numbers as $v_0 < \cdots < v_{L-1}$ and by making the following definitions:

$$\begin{aligned} v_{-2} &= v_{L-2} - p \ , \\ v_{-1} &= v_{L-1} - p \ , \\ v_L &= v_0 + p \ , \text{and} \\ v_{L+1} &= v_1 + p \ ,\ldots. \end{aligned} \tag{S11}$$

an expanded sequence is obtained satisfying $v_{-2} < v_{-1} < v_0 < \cdots < v_{L-1} < v_L < v_{L+1}$. For any $i = 0, \ldots, p-1$, we then define $\rho(i)$ to be the unique integer from $0 \ldots L-1$ satisfying the relations

$$v_{\rho(i)-1} < v_{\rho(i)} \leq i < v_{\rho(i)+1} < v_{\rho(i)+2} \ , \tag{S12}$$

which is well-defined as a result of the definitions in equation (S11). As a result of these definitions, $\rho(i)$ allows for the selection of appropriate data from the columns of M (stored in a permuted order from the sequencing of aggregate patterns) to implement piecewise interpolation with cyclic boundary conditions matching the indexing of Y (stored in non-permuted order).

With i fixed, four coefficients $h_0, \ldots, h_3$ are then computed:

$$[h_0 \ \ h_1 \ \ h_2 \ \ h_3] = H\left(v_{\rho(i)-1},\ v_{\rho(i)},\ v_{\rho(i)+1},\ v_{\rho(i)+2},\ i\right) \ , \tag{S13}$$

from which the ith row of $\widehat{W}$ is then found as:

$$\widehat{W}_{i,j} = \begin{cases} h_0 & \text{if } j = \sigma^{-1}(\ \rho(i) - 1) \\ h_1 & \text{if } j = \sigma^{-1}(\ \rho(i)\ ) \\ h_2 & \text{if } j = \sigma^{-1}(\ \rho(i) + 1) \\ h_3 & \text{if } j = \sigma^{-1}(\ \rho(i) + 2) \\ 0 & \text{otherwise} \end{cases} \ , \tag{S14}$$

where $j = 0 \ldots L-1$, and the arguments to σ^{-1} are each interpreted modulo L.

For any segment of scan data, the index a for the aggregate pattern corresponding to the first row of M is known at the time of reconstruction. In general, with $\widehat{W}$ pre-computed and stored in memory, the matrix W used for interpolation of M depends on a according to

$$W_{i,j} = \widehat{W}_{i-a,\,j} \ , \tag{S15}$$

where the index $i - a$ is interpreted modulo p.

Supplementary references

1 Bouchard, M. B. *et al.* Swept confocally-aligned planar excitation (SCAPE) microscopy for high speed volumetric imaging of behaving organisms. *Nat Photonics* **9**, 113-119 (2015).

2 Wang, Y. *et al.* High Speed Computational Ghost Imaging via Spatial Sweeping. *Sci Rep* **7**, 45325 (2017).

3 Zhang, K., Hu, J. & Yang, W. Deep compressed imaging via optimized pattern scanning. *Photonics Res.* **9**, B57-B70 (2021).

4 Candes, E. J. & Romberg, J. K. l1-magic: Recovery of Sparse Signals via Convex Programming. (2005). <https://candes.su.domains/software/l1magic/>.

5 Bian, L., Suo, J., Dai, Q. & Chen, F. Experimental comparison of single-pixel imaging algorithms. *J Opt Soc Am A Opt Image Sci Vis* **35**, 78-87 (2018).

6 Wang, Z., Bovik, A. C., Sheikh, H. R. & Simoncelli, E. P. Image Quality Assessment: From Error Visibility to Structural Similarity. *IEEE Trans. Image Process.* **13**, 600-612 (2004).

7 Vaz, P. G., Amaral, D., Requicha Ferreira, L. F., Morgado, M. & Cardoso, J. Image quality of compressive single-pixel imaging using different Hadamard orderings. *Opt. Express* **28**, 11666-11681 (2020).

Supplementary figures

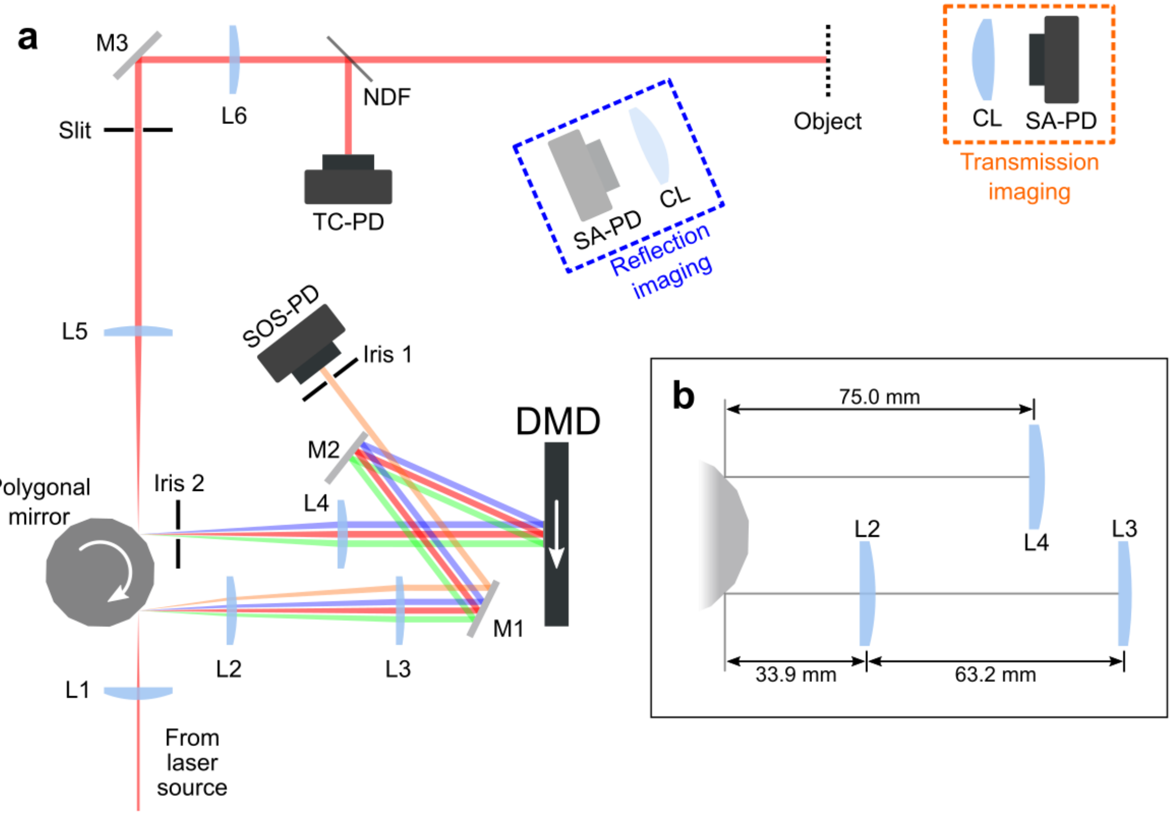

Supplementary Fig. 1.

Detailed system schematic of SPI-ASAP. a Scale illustration of system components. Four beam paths highlighting the scanning action are shown by the colors orange, blue, red, and green, in the order of appearance during scanning. **b** Closeup showing positions of lenses for beam scanning and de-scanning. CL: condenser lens. DMD: digital micromirror device. L1-L6: lenses. M1-M3: mirrors. NDF: neutral density filter. SA-PD: signal-acquisition photodiode. SOS-PD: start-of-scan photodiode. TC-PD: time-coding photodiode.

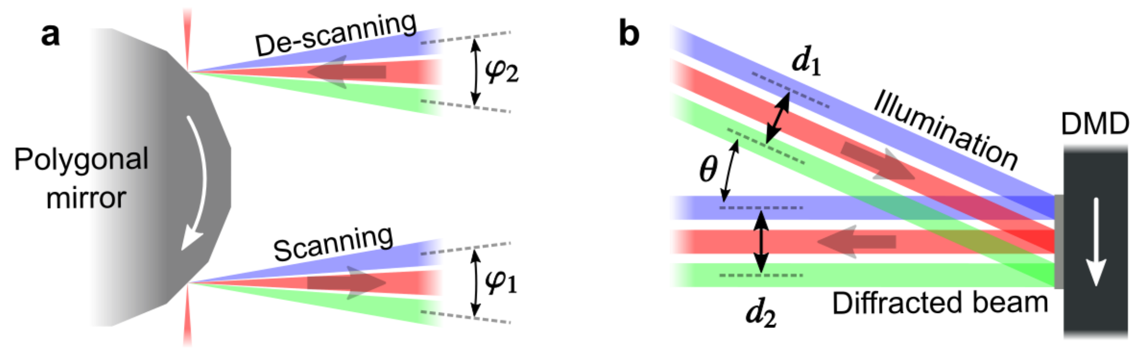

Supplementary Fig. 2.

Geometry of beam scanning. a Closeup of polygonal mirror reflections showing the correlation of beam angle necessary for scanning and de-scanning. **b** Closeup of DMD surface showing the effect of diffraction angle on lateral beam translation distances. In (**a**) and (**b**), white arrows indicate directions of polygonal mirror rotation and illumination scanning. Gray arrows show the laser beams' propagation direction.

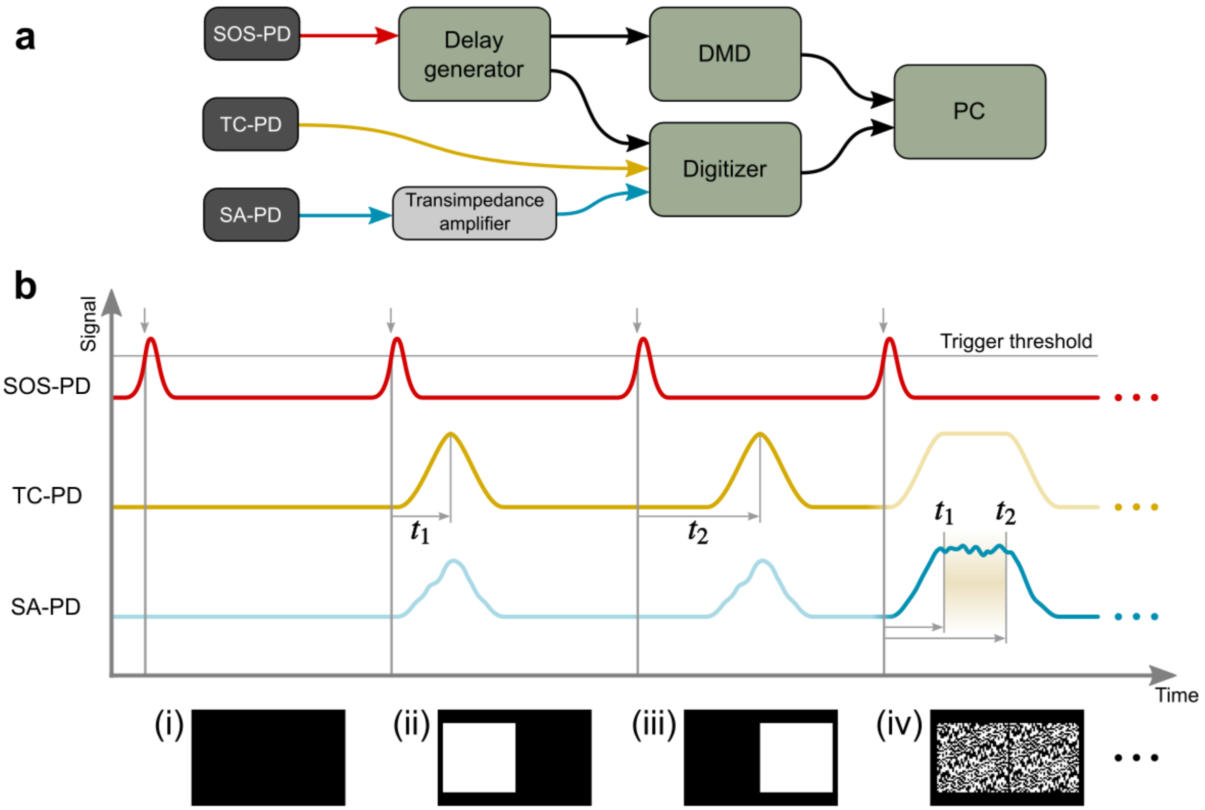

Supplementary Fig. 3.

Illustration of synchronization and data registration in SPI-ASAP. a Block diagram illustrating the paths of signals involved in timing and acquisition. **b** Schematic timing diagram illustrating synchronization and data registration. Insets show the initial frames of a DMD sequence for 41×43-pixel resolution imaging: (i) blank frame, (ii) initial pattern region, (iii) final pattern region, and (iv) the first aggregate pattern.

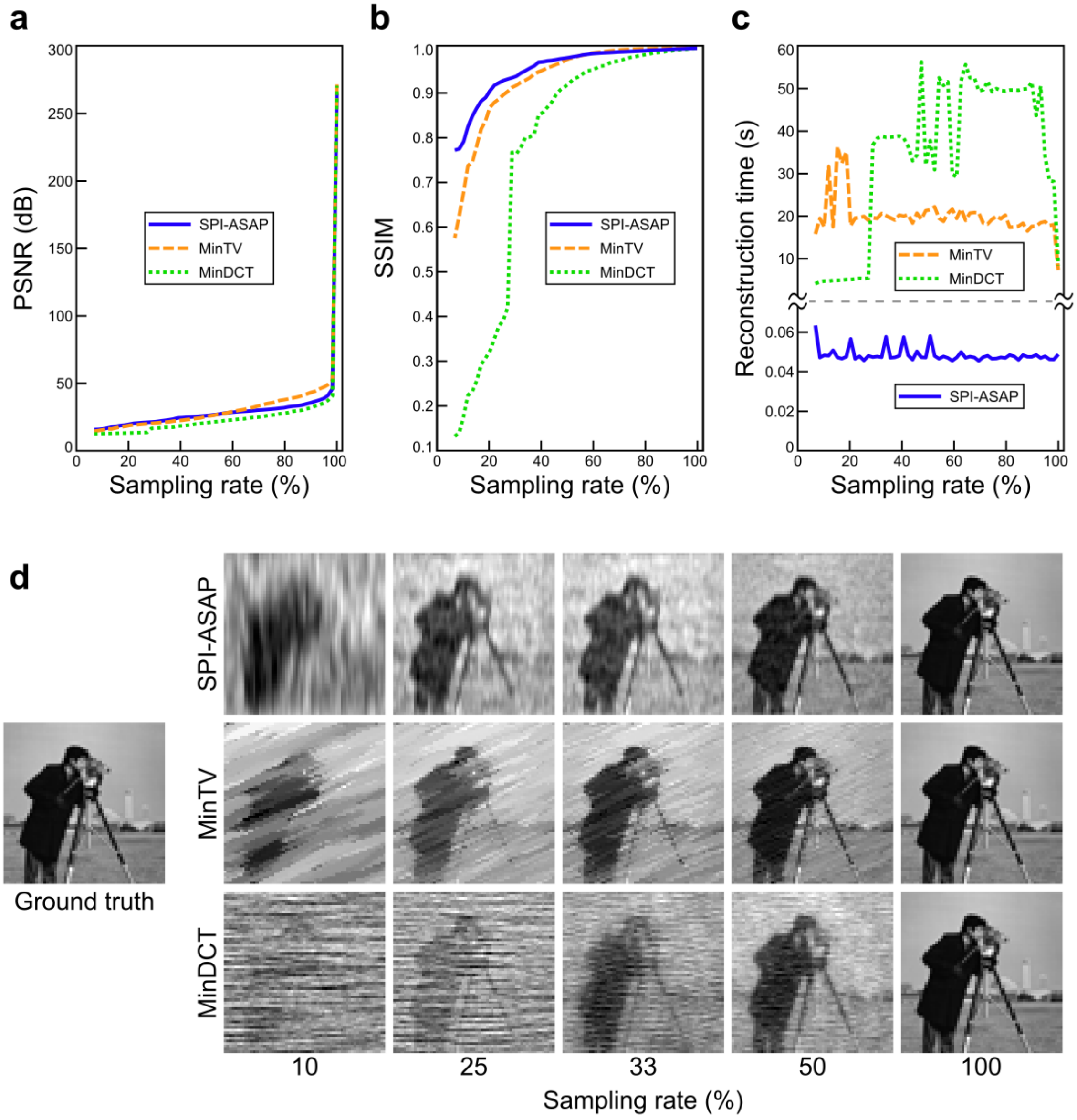

Supplementary Fig. 4.

Comparison of image reconstruction between SPI-ASAP and two widely used compressed-sensing algorithms. The ground truth is the “Cameraman” image with a frame size of 59×61 pixels. **a–c** Reconstruction quality evaluated by peak signal-to-noise ratio (PSNR) (a), structural similarity index (SSIM) (b), and running time (c) for SPI-ASAP, MinTV, and MinDCT plotted as a function of sampling rate for the ground truth image. Note that the vertical axis in (c) is discontinuous. **d** Visual comparison of a subset of reconstructed images.

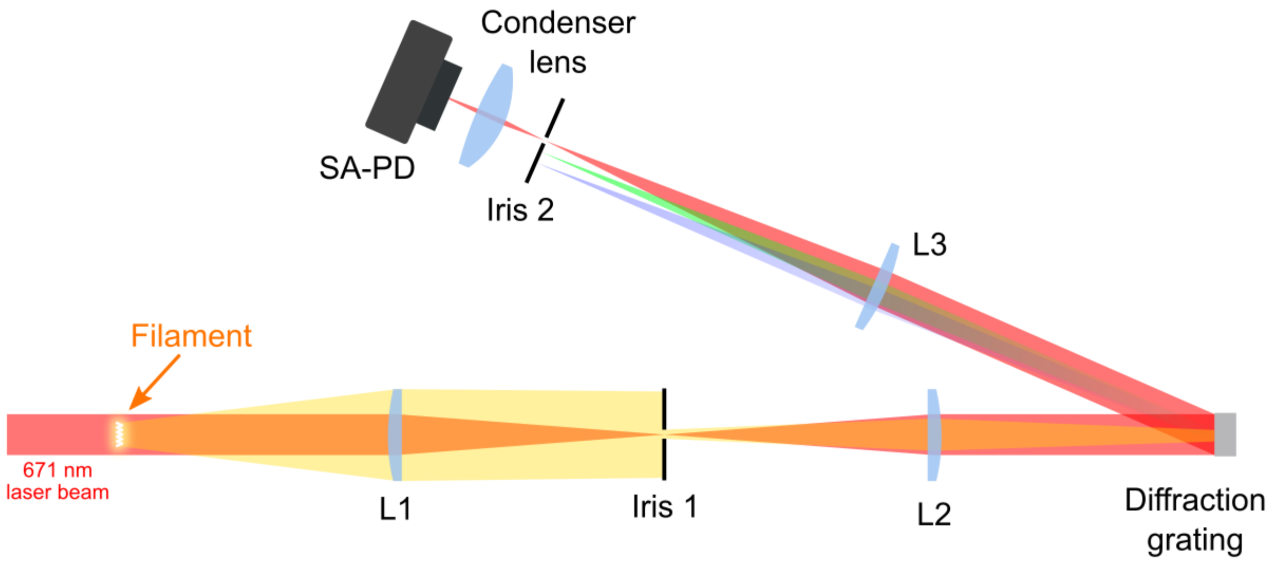

Supplementary Fig. 5.

Illustration of system setup for the high light interference imaging of incandescent filaments.

L1-L3: lenses. SA-PD: signal-acquisition photodiode.

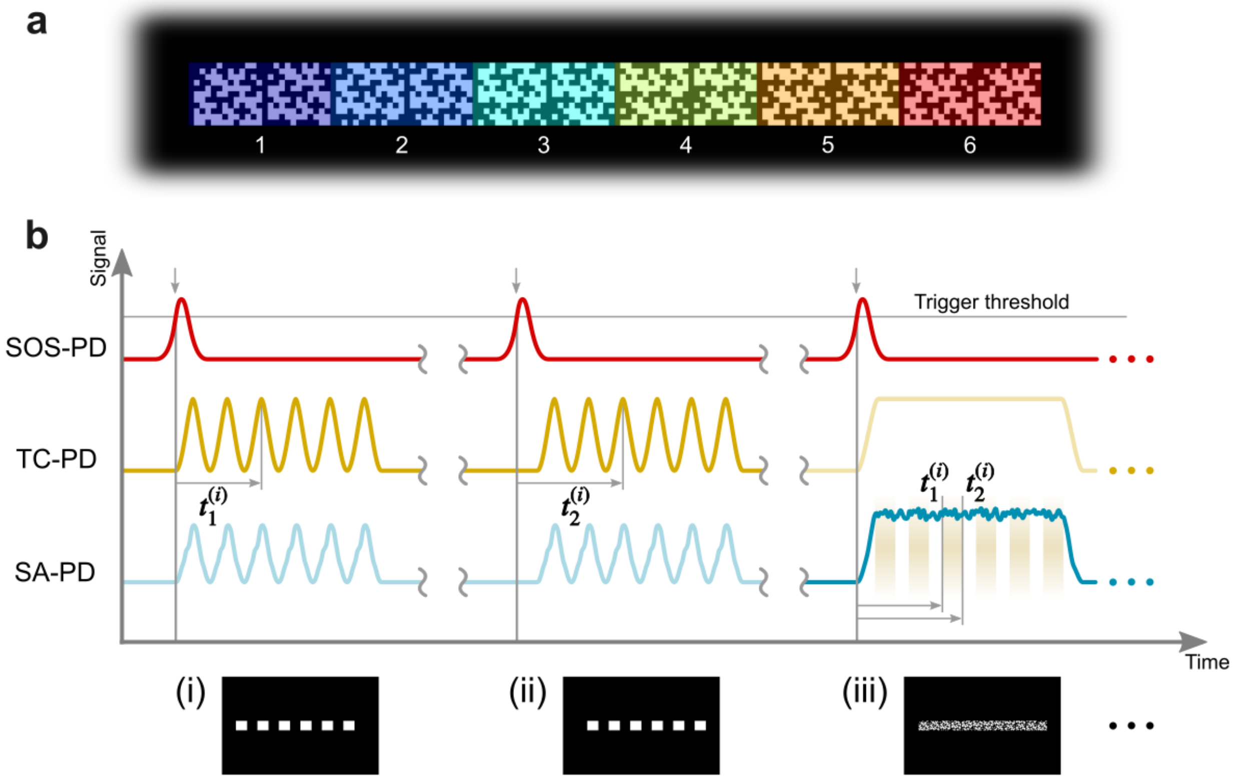

Supplementary Fig. 6.

Illustration of aggregate patterning and system timing for ultra-high-speed imaging at 12 kfps. a Multi-aggregate pattern scheme for 11×13-pixel resolution imaging consisting of six joint aggregate patterns (distinguished by the shaded colors). **b** Schematic timing diagram illustrating synchronization and data registration for the multi-aggregate pattern. Example waveforms corresponding to the display of initial (i) and final (ii) sub-regions on the mask, as well as (iii) the static multi-aggregate pattern.

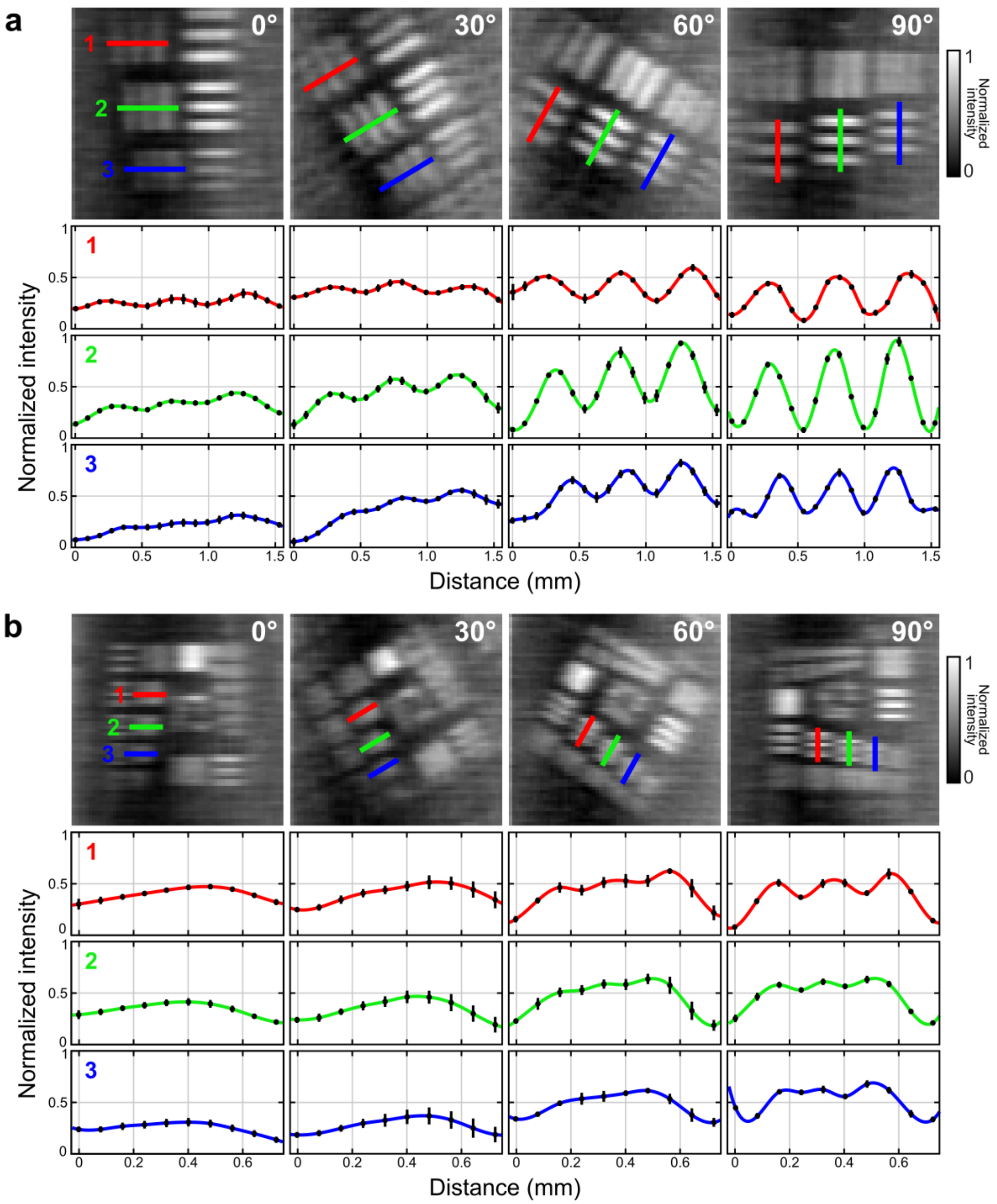

Supplementary Fig. 7.

Investigation of SPI-ASAP's spatial resolution. a Averaged image sequence and normalized intensity profiles of a resolution target (1951 USAF) rotated through 90°. Profiles 1–3 correspond to the vertical line pairs of Group 1 Elements 2–4, respectively. **b** As in **a**, but with profiles 1–3 corresponding to the vertical line pairs of Group 2 Elements 3–5, respectively. Error bar: standard deviation.

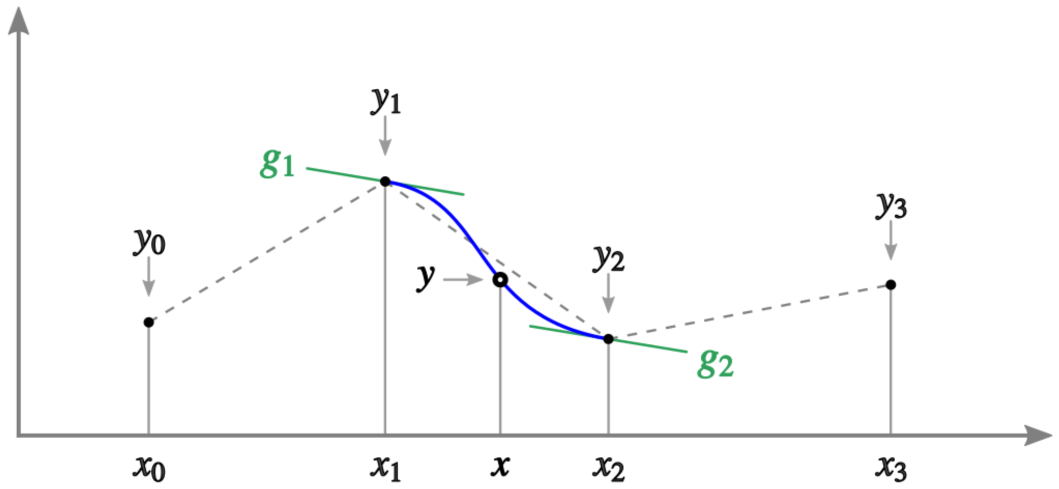

Supplementary Fig. 8.

Illustration of the parameters for cubic spline interpolation.

Supplementary Tables

	Scanning hardware	Mirror scanning rate	Modulation rate
DeCIOPS (Ref. [2])	1× resonant scanner<br>1× GM	Resonant Scanner: 8 kHz<br>GM: 125 Hz[(1)]	1.02 MHz[(2)]
Ref. [3]	2× GMs [(4)]	200 Hz	97 kHz
SPI-ASAP	1× polygonal mirror[(1)]	Multi-pattern: 6.37 kHz[(3)]<br>Single-pattern: 12 kHz[(4)]	Multi-pattern: 3.0–5.0 MHz<br>Single-pattern: 14.1 MHz

Notes:
[(1)]Computed according to the sampling of a 64×64 grid carried out in 4 ms
[(2)]Operation is synchronized
[(3)]Limited by the DMD's refresh rate
[(4)]Limited by the 16-facet polygon mirror

Supplementary Table 1.

Performance comparison of SPI-ASAP with state-of-the-art techniques in the same category.

Experiment	p	c
Fig. 6,<br>Supplementary Movie 6	11	2
Figs. 3a–c, 4b, 5b, 5e,<br>Supplementary Movies 1, 3, 4, 5	41	12
Fig. 4a,<br>Supplementary Movie 2,<br>Supplementary Movie 7 (Demonstrations 3, 4)	59	18
Supplementary Movie 7 (Demonstration 1)	71	21
Supplementary Movie 7 (Demonstration 2)	101	39

Supplementary Table 2.

Summary of parameters used to determine the sequencing of aggregate patterns.
