# Supplementary material for: Compressed ultrahigh-speed single-pixel imaging by swept aggregate patterns
Source: Nat Commun. 2022 Dec 22;13:7879. doi: 10.1038/s41467-022-35585-8 (PMC9780349; doi:10.1038/s41467-022-35585-8)
Supplement: Supplementary file 3 — Description of Additional Supplementary Files [file 41467_2022_35585_MOESM3_ESM.pdf]

Description of Additional Supplementary Files

Supplementary Movie 1:
Three reconstructed videos of an oscillating pendulum (1.53 s duration, 41×43 frame size), corresponding to the data presented in Fig. 3. Sampling rates (from left to right) are 100%, 50%, and 25%, with imaging speeds of 145 fps, 298 fps, and 598 fps, respectively.

Supplementary Movie 2:
Reconstructed video of the mechanical watch movement (2.20 s duration, 59×61 frame size, 103 fps framerate, 100% sampling), corresponding to the data presented in Fig. 4b.

Supplementary Movie 3:
Reconstructed video of a rupturing popcorn kernel (0.89 s duration, 41×43 frame size, 298 fps framerate, 50% sampling), corresponding to the data presented in Fig. 4e.

Supplementary Movie 4:
Reconstructed video of the current-induced failure of a sealed incandescent light bulb filament (1.06 s duration, 41×43 frame size, 298 fps framerate, 50% sampling), corresponding to the data presented in Fig. 5b.

Supplementary Movie 5:
Reconstructed video of the current-induced failure of an exposed incandescent light bulb filament (1.79 s duration, 41×43 frame size, 298 fps framerate, 50% sampling), corresponding to the data presented in Fig. 5e.

Supplementary Movie 6:
Reconstructed video of a 30-slot optical chopper rotating at 4800 RPM (9.83 ms duration, 11×13 frame size, 12.0 kfps framerate, 55% sampling), corresponding to the data presented in (from left to right) Fig. 6b–f.

Supplementary Movie 7:
Filmed demonstration of SPI-ASAP operating in real-time for a variety of scenes, frame sizes, and frame rates.
